# Truffle biogeography—A case study revealing ecological niche separation of different *Tuber* species

**DOI:** 10.1002/ece3.3017

**Published:** 2017-05-07

**Authors:** Milan Gryndler, Petr Šmilauer, Václav Šťovíček, Kristýna Nováková, Hana Hršelová, Jan Jansa

**Affiliations:** ^1^ Faculty of SciencesJan Evangelista Purkyně University in Ústí nad LabemÚstí nad LabemCzech Republic; ^2^ Laboratory of Fungal BiologyInstitute of MicrobiologyAcademy of Sciences of the Czech RepublicPragueCzech Republic; ^3^ Faculty of ScienceUniversity of South BohemiaČeské BudějoviceCzech Republic

**Keywords:** climate, environmental predictors, host tree identity, molecular detection, soil pH

## Abstract

Ecology of hypogeic mycorrhizal fungi, such as truffles, remains largely unknown, both in terms of their geographical distribution and their environmental niches. Occurrence of true truffles (*Tuber* spp.) was therefore screened using specific polymerase chain reaction (PCR) assays and subsequent PCR amplicon sequencing in tree roots collected at 322 field sites across the Czech Republic. These sites spanned a wide range of climatic and soil conditions. The sampling was a priori restricted to areas thought to be suitable for *Tuber* spp. inasmuch as they were characterized by weakly acidic to alkaline soils, warmer climate, and with tree species previously known to host true truffles. Eight operational taxonomic units (OTUs) corresponding to *Tuber aestivum*,* T. borchii*,* T. foetidum*,* T. rufum*,* T. indicum*,* T. huidongense*,* T. dryophilum*, and *T. oligospermum* were detected. Among these, *T. borchii* was the OTU encountered most frequently. It was detected at nearly 19% of the sites. Soil pH was the most important predictor of *Tuber* spp. distribution. *Tuber borchii* preferred weakly acidic soils, *T. foetidum* and *T. rufum* were most abundant in neutral soils, and *T. huidongense* was restricted to alkaline soils. Distribution of *T. aestivum* was mainly dictated by climate, with its range restricted to the warmest sites. Host preferences of the individual *Tuber* spp. were weak compared to soil and climatic predictors, with the notable exception that *T. foetidum* appeared to avoid oak trees. Our results open the way to better understanding truffle ecology and, through this new knowledge, also to better‐informed trufficulture.

## 1 Introduction

Ecology of hypogeic fungi is still only imperfectly known, mainly because of their cryptic lifestyle and erratic formation of fruiting bodies (Trappe et al., 2009). This precludes easy monitoring of their incidence and/or abundance throughout the year. This difficulty is further compounded by the fact that some of the hypogeic fungi, such as true truffles (*Tuber* spp.), are trophically dependent on certain host plants, forming so‐called mycorrhizal symbiosis with their roots and involving themselves in an exchange of nutrients and carbon with their hosts. This means their incidence is further restricted by the range of their (potential) hosts. Myths and anecdotal knowledge abound, and these are intensified by the fierce competition among lawful and unlawful truffle hunters to secure the marketable fruiting bodies. Yet, with the advent of cultivation‐independent detection and quantification of fungi, including of truffles (Gryndler, Trilčová, et al., 2013; Tedersoo et al., 2014), in environmental samples, our capacity has dramatically increased to examine their incidence, the environmental constraints of their geographical distribution, and the dynamics of these organisms' development (Gryndler et al., 2015). Such information is important not only for the sake of satisfying scientific curiosity, but also to facilitate exploitation of this rare commodity that is in high demand and to identify environmental conditions conducive to truffle cultivation (trufficulture). Detailed knowledge on the evolution and biogeography of different truffle groups, species, and genotypes is desirable for understanding their current geographical distribution (Bonito et al., 2013) and the mechanisms of their persistence in ecosystems (Zotti et al., 2013). This knowledge will be useful in monitoring and/or preventing human‐induced introgression of alien truffle species in new ranges that potentially could contaminate indigenous truffle communities (Bonito, Trappe, Donovan, & Vilgalys, 2011; Murat, Zampieri, Vizzini, & Bonfante, 2008). Much remains to be explored in relation to true truffles, including the host ranges (Gryndler, 2016; Gryndler, Černá, Bukovská, Hršelová, & Jansa, 2014), environmental determinants, and geographical distribution of the various truffle species (Bonito, Gryganskyi, Trappe, & Vilgalys, 2010; Serrano‐Notivoli, Incausa‐Gines, Martin‐Santafe, Sanchez‐Duran, & Barriuso‐Vargas, 2015; Splivallo et al., 2012). The current lack of such information is mainly due to a paucity of dedicated, large‐scale studies employing molecular detection to address truffle communities in soils (Leonardi et al., 2013; Taschen et al., 2016). This means most of the current knowledge is reliant on information from fruiting body collections or spatially restricted molecular studies (e.g., Berch & Bonito, 2016; Marjanovic, Grebenc, Markovic, Glisic, & Milenkovic, 2010; Pomarico, Figliuolo, & Rana, 2007).

As explained by Streiblová, Gryndlerová, and Gryndler (2012), the Czech Republic is a country with an historic tradition of truffle collection and marketing. Truffles lost their importance as a market commodity at the end of 19th century, however, and even the literature reports on their occurrence in historic times are limited (Streiblová, Gryndlerová, Valda, & Gryndler, 2010). The oldest reports are summarized by Streiblová et al. (2012), while more recent records have been presented by Klika (1927), Vacek (1947a,b, 1948, 1950), Šebek (1987, 1992), and Valda (2009). Occasional newer finds of truffle ascocarp described in the above references indicate that at least eight species of true truffles are indigenous to the Czech Republic (Valda, 2009). The rarity of records resulted in declaring one of the economically most valuable truffle species, *Tuber aestivum*, as a critically endangered species in the Czech Republic and in its protection by law (Kotlaba, 1995; Šebek, 1987). A question appears whether this species is truly so rare or if its abundance in ecosystems is underestimated due to its hypogeous nature and resulting difficulties in finding the ascocarps. The occurrence of *Tuber* spp. in general and of *T. aestivum* in particular should thus be addressed by systematic screening of multiple field sites while using the currently available arsenal of cultivation‐independent methods that are based on molecular detection of organisms in soil (Bonito et al., 2010; El Karkouki, Murat, Zampieri, & Bonfante, 2007). The great diversity of soil and climatic conditions at spatial scales suitable for single‐study sample collection make the territory of Czech Republic particularly suitable as a model area for addressing ecological niche separation of different *Tuber* spp. as well as to challenge the approaches currently available for molecular detection of the various truffle species.

Molecular tools that are currently being used in detection of different *Tuber* spp. mainly rely on polymerase chain reaction (PCR) using species‐specific primers (Amicucci, Zambonelli, Giomaro, Potenza, & Stocchi, 1998; Bertini et al., 2006; Bonito, 2009; Gryndler et al., 2011; Iotti et al., 2007; Mello, Cantisani, Vizzini, & Bonfante, 2002; Mello, Garnero, & Bonfante, 1999; Séjalon‐Delmas et al., 2000). Considerable efforts have been invested, too, into developing primers that would enable detection of the entire *Tuber* genus (Bertini et al., 1999; Zampieri, Mello, Bonfante, & Murat, 2009). That would potentially provide much more information about the diversity of *Tuber* spp. at individual field sites, and particularly if it were used in combination with the detection of specific sequence motifs in the amplicons, such as through dot‐blot hybridization (El Karkouki et al., 2007) or massively parallel amplicon sequencing (Mello et al., 2011; Tedersoo et al., 2014).

To improve our understanding of true truffle ecology, and with particular reference to central Europe, we conducted large‐scale field sampling and molecular detection of *Tuber* spp. in roots of potential host trees using two parallel PCR approaches: targeting *Tuber* spp. at genus level and *T. aestivum* at species level. The sampling was a priori restricted to warmer parts of the country which are suitable to support the occurrence of *T. aestivum*, the economically most important *Tuber* sp. domestic to the Czech Republic (Stobbe et al., 2013), and the sampling avoided particularly acidic soils. Soil samples containing roots were only collected under tree species known to establish mycorrhizal symbiosis with truffles. Host plant identity at the individual sites was recorded, as were soil properties and climatic parameters, to allow for a posteriori testing of true truffles' niches along large geographical and environmental gradients. In particular, we asked the following questions:

1. Which *Tuber* spp. can be detected using the PCR approaches described above in the field root samples? Are economically important species such as *T. aestivum* among them?
2. Is the number of *Tuber* OTUs detected by the PCR approaches within a region comparable to the number of *Tuber* spp. recorded as ascocarps in the same region?
3. What are the environmental determinants (if any) of the occurrence of different *Tuber* spp.?

## 2 Materials and Methods

### 2.1 Sample collection

**Figure 1:**
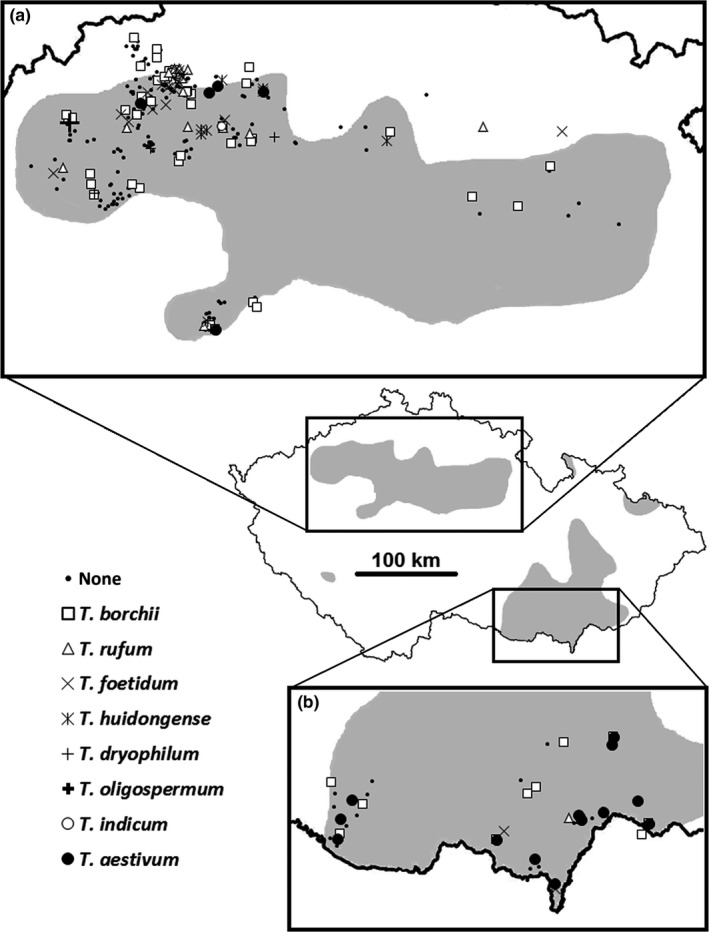
Geographical distribution of the *Tuber* species as detected by specific PCR assays in this study. Gray‐shaded areas indicate northern and central Bohemia (a) and southern Moravia (b) regions with predominant occurrence of weakly acidic to alkaline soils (according to data provided by the Research Institute for Soil and Water Conservation, Prague—Zbraslav, Czech Republic, www.vumop.cz), to which most of the sampling efforts were directed

Soil cores were collected at 322 sites beneath trees of species known as *Tuber* hosts (*Quercus* spp., *Carpinus betulus*,* Corylus avellana*,* Tilia spp*., *Pinus nigra*, or *Fagus sylvatica*). Sites were chosen randomly within predetermined warmer climatic regions of the Czech Republic having weakly acidic, neutral, or alkaline soils and where *Tuber* ascocarps have occasionally been recorded in the past (shaded areas in Figure 1). The sampling strategy was based on randomly generated positions of potential sampling sites, and sampling was carried out only if a suitable potential host was present at the site. Sampling density was further increased in the north‐western part of the Czech Republic because of its diverse terrain and, at the same time, calcareous (limestone) bedrock, which is considered suitable for *Tuber* species.

The cores were taken at depth 0–10 cm using cylindrical plastic corers 25 mm in diameter. At each of the field sites, five cores were taken and transported to the laboratory within 2 days from sampling. In the laboratory, the soil was pressed out of the corers and pooled per sampling site, and roots were picked out using forceps. The roots were then washed with sterile tap water to remove residual soil particles and frozen for subsequent analyses. The pooled soil was then well mixed for each sample and dried at room temperature.

### 2.2 Soil analyses

Soil samples were suspended in deionized water (1:2, w:v), and pH was measured in the slurry after 30 min of equilibration. Electrical conductivity was measured in the liquid above the slurry using an OK102/1 conductivity meter (Radelkis, Budapest, Hungary). Saturation concentration of Ca^2+^ was measured using an ion‐selective electrode (Monokrystaly, Turnov, Czech Republic) in the soil water extracts after 12 hr of incubation with shaking at room temperature and after pH of the extracts had been adjusted to 7.0 (Sochorová et al., 2016).

Soil water extracts were further used to measure trophic potential of the soil (in relation to the content of mineral nutrients necessary for *Chlorella kessleri* growth) as described in Gryndler, Soukupová et al. (2013) and in the Supplementary information, section “Estimation of soil trophic potential.”

### 2.3 DNA extraction and PCR

The roots containing ectomycorrhizae were used for DNA extraction. To this end, fresh roots (ca 50 mg samples) were extracted with CTAB‐Tris extraction buffer and purified using the glass milk procedure as described in Gryndler et al. (2011).

Presence or absence of the various *Tuber* spp. was detected by nested PCR assays. Before the products of the first PCR were used as templates in the second PCR, they were always 100× diluted. To assess the presence of *T. aestivum*, a nonspecific first PCR was directed to the ITS region of the nuclear rRNA cassette (forward primer NSI1, reverse primer NLB4; Martin & Rygiewicz, 2005). Thereafter, the second, *T. aestivum*‐specific, PCR used forward primer Tu1sekvF and reverse primer Tu2sekvR (Gryndler et al., 2011). Other *Tuber* spp. were detected by selective amplification of the β‐tubulin gene using first PCR with forward primer Bt2a and reverse primer Bt2b (Glass & Donaldson, 1995), followed by a second PCR employing genus *Tuber‐*specific PCR using forward primer tubtubf and reverse primer elytubr (Zampieri et al., 2009).

The PCR (25 μl volume) was always composed of 12.5 μl of 2 ×  Combi‐PPP master mix (Top‐Bio, Prague, Czech Republic; contains hot start‐Taq DNA polymerase, 5 mmol/L MgCl_2_, buffer, deoxyribonucleotides and gel loader), 0.5 μl 10 μmol/L forward primer, 0.5 μl 10 μmol/L reverse primer, 0.5 μl DNA template, and 11 μl PCR‐grade water.

The thermal cycling programs were as follow:

Detection of *T. aestivum*, first PCR: 95°C, 4 min, 29 cycles (95°C, 60 s; 52°C, 45 s; 72°C, 120 s), 72°C for 5 min;
Detection of *T. aestivum*, second PCR: 95°C, 4 min, 40 cycles (95°C, 40 s; 59°C, 40 s; 72°C, 40 s), 72°C for 5 min;
Detection of other *Tuber* spp., first PCR: 95°C, 4 min, 35 cycles (94°C, 45 s; 50°C, 45 ; 72°C, 90 s), 72°C for 5 min; and
Detection of other *Tuber* spp., second PCR: 94°C, 4 min, 25 cycles (94°C, 45 s, 50°C, 45 s, 72°C, 90 s), 72°C for 5 min.

Products of the second PCR detecting *T. aestivum* were subsequently digested by Tail restriction endonuclease to exclude cross‐amplification of closely related *Tuber mesentericum* (Gryndler et al., 2011) and visualized using agarose gel electrophoresis. Products of the second PCR detecting other *Tuber* spp. were visualized by agarose gel electrophoresis directly. Negative controls without template DNA were always used in each of the PCR assays to rule out contaminations of the components of the PCR mixture.

Products of nested PCR were then purified by isopropanol precipitation and unidirectionally (Sanger) sequenced using the primers Tu1sekvF (ITS region) or tubtubf (β‐tubulin gene). The sequences were manually edited and then identified by BLASTN search in the GenBank database. All the readable sequences were subsequently submitted to GenBank and are freely accessible under the accession numbers listed in Table 1.

**Table 1:** Operational taxonomic units (OTUs) delineated among the sequences generated in this study and named for the best GenBank hit corresponding to *Tuber* spp

OTU	No. of sequences (% of total sequence number)	Mean similarity (%) with the best GenBank hit	GenBank accession numbers of newly generated sequences
*T. borchii*	60 (52.2)	98.48 ± 0.85	KX303485, KX303487, KX303492, KX303493‐KX303496, KX303503‐KX303505, KX303507, KX303509, KX303513‐KX303516, KX303518, KX303522, KX303526, KX303527, KX303531, KX303532, KX303534, KX303535, KX303537, KX303538, KX303540‐KX303543, KX303546, KX303547, KX303551‐KX303557, KX303559, KX303561, KX303563‐KX303571, KX303573, KX303574, KX303577, KX303581‐KX303583, KX303586, KX303589‐KX303591
*T. rufum*	15 (13.0)	97.43 ± 1.72	KX303486, KX303490, KX303506, KX303508, KX303512, KX303524, KX303528, KX303536, KX303548, KX303549, KX303558, KX303572, KX303579, KX303584, KX303588
*T. foetidum*	19 (16.5)	98.87 ± 0.51	KX303488, KX303489, KX303491, KX303497‐KX303499, KX303500‐KX303502, KX303517, KX303519, KX303520, KX303523, KX303530, KX303545, KX303550, KX303562, KX303575, KX303580
*T. huidongense*	8 (7.0)	96.24 ± 0.76	KX303510, KX303511, KX303533, KX303560, KX303576, KX303578, KX303585, KX303587
*T. dryophilum*	3 (2.6)	98.40 ± 0.62	KX303521, KX303539, KX303544
*T. oligospermum*	1 (0.9)	98.2	KX303525
*T. indicum*	1 (0.9)	96.5	KX303529
*T. aestivum*	8 (7.0)	99.51 ± 0.30	KX303477‐KX303484

Mean value of sequence similarity with the best GenBank hit is shown ±*SD*.

### 2.4 Data analysis

All DNA sequences obtained in this study were assigned to operational taxonomic units (OTUs) corresponding to the different *Tuber* spp. according to the best GenBank hit identity.

Reliability of the categorization based on the β‐tubulin gene was then checked by phylogenetic analysis using the maximum‐likelihood method based on the Tamura–Nei model (Tamura & Nei, 1993). Before the analysis, the sequences KX303525, KX303544, KX303548, KX303521, KX303498, KX303517, KX303520, KX303523, KX303526, KX303545, KX303588, KX303493, and KX303585 were excluded due to short alignment coverage with other sequences. Remaining sequences were trimmed to the length of 235 nucleotide positions corresponding to nucleotides 91–321 of the sequence KX303590. The bootstrap consensus tree was inferred from 5,000 replicate trees. The tree was rooted on the *Penicillium chrysogenum* KC339225 and *Helvella ephippium* JN391114 sequences. Sequences FJ560929 (*T. borchii*), FN252811 (*T. dryophilum*), DQ336309 (*T. rufum*), GU979146 (*T. huidongense*), FN256291 (*T. foetidum*), and GU979181 (*T. indicum*) were included as *Tuber* species references. The analysis was performed using Mega 7 software (Kumar, Stecher, & Tamura, 2016).

Incidence of the various *Tuber* OTUs (after omitting two OTUs with just a single occurrence each in the entire data set) was related to the identity of potential host trees present at the sampling sites, to soil chemical properties, and to such climatic parameters as mean annual temperature, mean winter temperature, annual precipitation, and precipitation balance. The values of climatic parameters were obtained from publicly available maps produced by the Czech Hydrometeorological Institute, Prague. First, we tested individual effects of each of these three predictor groups on the *Tuber* incidence data; subsequently, we reduced each of the groups by a stepwise selection of the most significant predictors. During the stepwise selection, the significance levels were transformed into false discovery rates (FDR; Verhoeven, Simonsen, & McIntyre, 2005). The relative explanatory power of the three predictor groups was then compared by employing a variation partitioning approach using canonical correspondence analysis (CCA) constrained ordination with a Monte Carlo permutation test (Canoco 5 software; ter Braak & Šmilauer, 2012).

Further, to explore the effects of individual environmental predictors on the occurrence of particular *Tuber* spp. across all 322 samples, we used generalized linear models with numerical explanatory variables (i.e., the predictors) expressed as second‐order polynomials, fitted in the R software, version 3.2.2 (R Foundation for Statistical Computing, Vienna, Austria, https://www.r-project.org). The choice of polynomial terms provides compatibility of the underlying model of abundance change along environmental predictors with the unimodal model assumed by the multivariate CCA (Ter Braak, 1985) used for the whole community. Similar to the multivariate approach, for each OTU (excluding three OTUs with less than four occurrences each in the data set), we first compared a model containing all predictors from a particular predictor group with the null model using a likelihood ratio test. Only if the model turned out to be significant did we identify the predictors with significant partial effects again using the likelihood ratio test and adjusting the estimated type I error probabilities by transforming them into FDR values. Selected significant polynomial terms were checked for the implied shape of the fitted effect, and in the case of a curve with a minimum (rather than one with a clear optimum, compatible with the unimodal niche model), a linear term was fitted and tested instead.

Because soil pH was identified to be the most significant predictor of *Tuber* spp. occurrence among all tested environmental predictors, we analyzed it further. For each OTU with more than three occurrences in the data set, a generalized linear model was fitted, starting with the null hypothesis (i.e., no significant change with pH), testing first a linear model, and then testing a second‐order polynomial (unimodal) model against the linear, if significant. Binomial distribution of the OTUs incidence data was assumed, and the logit link function was used in the analysis. Determined were the amount of explained variation and estimate of optimum pH values, 95% confidence intervals of the pH optima, as well as the tolerance ranges.

Descriptive statistics and value ranges for climatic parameters and soil properties are given in Table S1.

## 3 Results

### 3.1 Frequency of *Tuber* spp. detection in soil samples

Of the 322 sites included in this study, 107 sites showed the presence of *Tuber* spp. based on PCR amplification of the β‐tubulin gene with *Tuber*‐specific primers. These records could subsequently be confirmed by sequencing (Table 2). Another 24 positive signals based on the PCR amplification were mixed sequences and thus unreadable by Sanger sequencing. Still another two positive PCR amplifications were false positives inasmuch as the sequences obtained were similar to *Helvella ephippium* β‐tubulin sequence JN392114 rather than being affiliated with *Tuber* spp.

**Table 2:** Summary of polymerase chain reaction (PCR)‐based detections of the different *Tuber* species in roots collected at the different sampling sites

Target	No. of positive PCR results	No. of negative PCR results	No. of positive low‐quality PCR results	No. of false‐positive PCR results
ITS—*Tuber aestivum*	8	302	10	2
ITS—other *Tuber* spp.	0	322	0	0
β‐Tubulin—other *Tuber* spp.	107	189	24	2
β‐Tubulin—*Tuber aestivum*	0	322	0	0

Results were obtained either with nested PCR specific to *T. aestivum* (targeting the internal transcribed spacer [ITS] region of the ribosomal DNA) or for several other *Tuber* species (targeting the β‐tubulin gene). The numbers of low‐quality and false‐positive PCR results indicate the numbers of unreadable sequences (most probably because of mixed sequence types) and sequences that were identified as belonging to fungi other than *Tuber* spp., respectively.

Eight positive PCR amplifications were recorded for *Tuber aestivum* (ITS region) among the samples and which could subsequently be verified by sequencing. Another 10 PCR amplicons with the *T. aestivum*‐specific primers were unreadable by Sanger sequencing (Table 2). Nevertheless, those amplicons displayed Tail restriction profiles corresponding to *T. aestivum* (not shown) and thus were retained in the data set. Two false‐positive signals were recorded with *T. aestivum*‐specific primers: A sequence from one amplicon showed similarity to *Sphaerosporella sp*. JQ711781 and the other similarity to *Trechispora invisitata* KP814425.

Seven sites showed positive *Tuber*‐specific β‐tubulin signal simultaneously with ITS signal of *T. aestivum,* with the latter either verified or unverified by sequencing (see Table S2 for details). At the same time, amplicons generated with *Tuber*‐specific primers targeting the β‐tubulin gene that returned low‐quality (illegible) sequences constituted approximately 22% of positive signals that could unequivocally be verified by sequencing (Table 2).

### 3.2 OTU delineation

The sequences generated from β‐tubulin amplicons were first assigned to the various *Tuber* spp. by direct comparison with GenBank, taking the best hit as the closest relative (Table 1). Using this approach, we identified eight distinctly named OTUs. Other than best hits, however, the different sequences showed similarity to many other *Tuber* spp. in a number of cases, albeit with lower similarity scores. Therefore, we further scrutinized the identities of the various OTUs for reliability and consistency throughout our sequence set.

**Figure 2:**
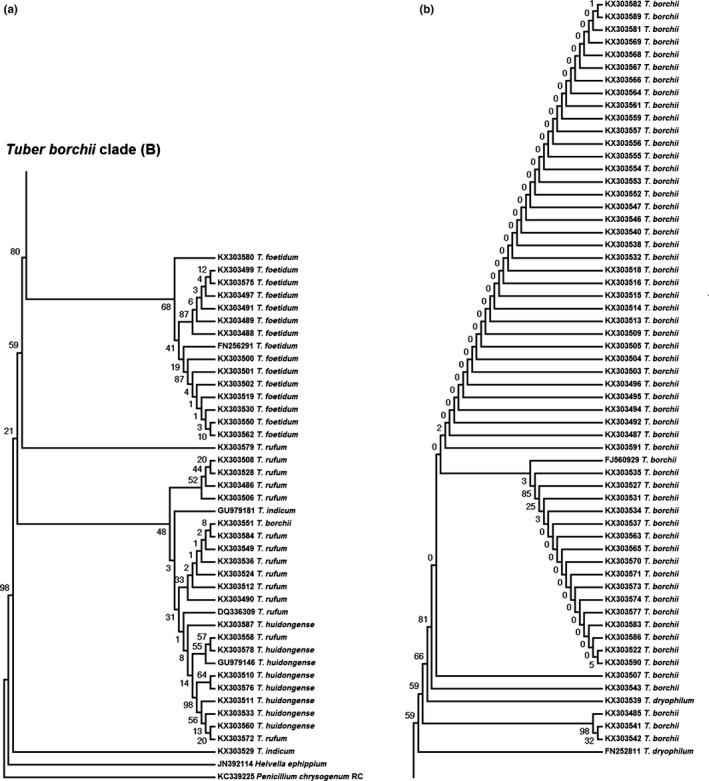
Maximum‐likelihood bootstrap consensus cladogram describing the evolutionary relatedness of the β‐tubulin gene sequences obtained from *Tuber* spp. using the specific PCR assays described in this study (a), with the “*T. borchii* clade” presented separately (b). The tree with the highest log likelihood (−1,271.9) is shown. The percentage of replicate trees (*n* = 5,000) in which the associated taxa clustered together in the bootstrap test are shown next to the branches. Sequence identifiers with first two letters other than “KX” indicate reference sequences downloaded from GenBank

To this end, phylogenetic relationships between the different β‐tubulin sequences were analyzed using the maximum‐likelihood bootstrap method (Figure 2). This analysis returned a very well‐supported clade of *T. borchii* (Figure 2b), which was conserved in 81% of the generated tree replicates. The sequences belonging to this clade were most similar to GenBank sequences FN252810 and FJ560925. Only four of 60 sequences previously assigned to *T. borchii* fell outside of this clade, although three of those outliers were nevertheless located very close to it and were intermixed with sequences of *T. dryophilum*. Those sequences showed the greatest similarity with GenBank *T. borchii* sequence FJ560919. A single distant outlier, the sequence KX303551, was similar to GenBank sequence FN252810, which was the best hit for many members of the main *T. borchii* clade cited above.

Another homogeneous and well‐supported (68% of all 5,000 generated trees) clade consisted of sequences similar to *Tuber foetidum* (Figure 2a). All the sequences were similar to GenBank sequence FN256291 that also served as reference for this OTU. The third notable clade, supported at 48%, was the clade containing sequences originally attributed to *T. rufum* and *T. huidongense*. Within the clade, the two OTUs were poorly separated. The sequence attributed to *T. indicum* did not associate with its reference sequence GU979181.

The mean similarity of β‐tubulin sequences attributed to *T. borchii* and *T. foetidum* to best GenBank hits was well above 98% and was surpassed only by the similarity of *T. aestivum* ITS sequences to their GenBank best hits, which exceeded 99% (Table 1). Similarities of sequences attributed to *T. rufum* and *T. huidongense* to their GenBank references were lower, reaching 97% and 96%, respectively. Although well separated from other sequences, the sequence attributed to *T. indicum* showed relatively poor similarity to its best GenBank hit, reaching just 96%.

### 3.3 Environmental predictors of *Tuber* spp. incidence

**Figure 3:**
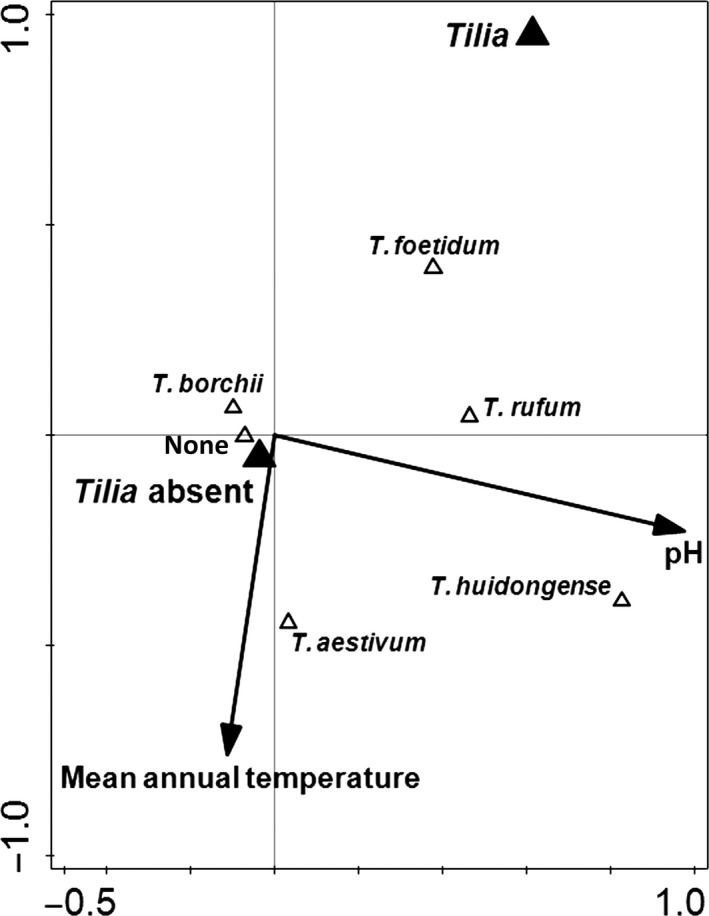
Canonical correspondence analysis (CCA) biplot showing association of the different *Tuber* species with selected environmental predictors. The predictors together explain (based on the first two canonical axes) 5.9% of the total variation in *Tuber* spp. incidence data. Label “None” indicates samples where no *Tuber* spp. was detected

Using the CCA approach (Table 3), we found that the predictors from each of the three groups (host species, climatic parameters, and soil properties) explained 6.3% of total variation in the incidence of *Tuber* OTUs across the different sampling sites (Table 3). Further analysis indicated that the effects attributed to each of the predictor groups could be explained by a single predictor within each of the groups, namely the presence of *Tilia* spp., mean annual temperature, and pH, respectively (Figure 3). Within each of the predictor groups, there was one additional predictor with a significant independent (simple) effect, namely presence of *Quercus* spp., mean winter temperature, and soil conductivity, respectively. After selecting the main explanatory predictor into the model in each case, however, the effect of the second predictor within each such group was rendered nonsignificant. These results supported the choice of carrying out the variation partitioning using only a single predictor for each of the groups.

**Table 3:** Variability of the incidence of the different *Tuber* species at the different sampling sites as explained by presence or absence of *Tilia*, mean annual temperature and soil pH, the selected predictors among the host plant, climatic parameters, and soil properties predictor groups, respectively

Variability fraction	% of explained variation	% of total variation
Host tree species (*Tilia*)	17.2	1.1
Climate (mean annual temperature)	24.1	1.5
Soil (pH)	57.0	3.6
Host tree and climate	−1.3	−0.1
Climate and soil	−0.2	−0.0
Soil and host tree species	3.7	0.2
Host tree and climate and soil	−0.3	−0.0
Total explained	100.0	6.3

The analysis was performed using canonical correspondence analysis (CCA). The variability fractions including “&”represent the variation explained jointly by two or three groups of predictors. Negative values for explained variation result from working with nonadditive adjusted explained variation (*R*_adj_^2^) and should be interpreted as zeros.

Variation partitioning (Table 3) showed that pH had the strongest explanatory power with respect to *Tuber* spp. incidence at the different sampling sites. It explained more variation than did the other two parameters combined. This is also reflected in Figure 3, where the horizontal axis is virtually coincident with the gradient of pH, increasing from left to right.

The results of exploring the effects of environmental parameters by means of generalized linear models on the presence of individual *Tuber* OTUs at the different sampling sites are summarized in Table 4. These analyses showed that host tree identity had almost no significant effect on the *Tuber* OTU incidence (with the single exception of *T. foetidum* avoiding *Quercus* spp.), whereas climatic parameters such as winter temperature and precipitation showed a unimodal relationship with the incidence of the *T. huidongense* OTU. Further, positive correlation with mean annual temperature was noted for the *T. aestivum* OTU.

**Table 4:** Summary of significant effects in generalized linear models (where the effects of numeric climatic parameters and soil properties are expressed as second‐order polynomials or linear terms) predicting the probability of occurrence for individual operational taxonomic units (OTUs) representing different *Tuber* species

OTU	Hosts	Climatic parameters	Soil properties
*T. borchii*	n.s.	n.s.	Negative relation with soil conductivity ($\chi_{1}^{2}=6.63$, *p*_adj_ = .010) Unimodal relation with soil pH ($\chi_{1}^{2}=12.84$, *p*_adj_ = .007)
*T. rufum*	n.s.	n.s.	Unimodal relation with soil pH ($\chi_{1}^{2}=23.00$, *p*_adj_ < .001)
*T. foetidum*	avoids *Quercus* ($\chi_{1}^{2}=8.43$, *p*_adj_ = .018)	n.s.	Unimodal relation with soil pH ($\chi_{1}^{2}=24.86$, *p*_adj_ < .001)
*T. huidongense*	n.s.	Unimodal relation with winter temperature (prefers higher values) ($\chi_{1}^{2}=8.04$, *p*_adj_ = .050) Unimodal relation with precipitation (prefers lower values) ($\chi_{1}^{2}=7.37$, *p*_adj_ = .050)	Unimodal relation with soil pH ($\chi_{1}^{2}=12.29$, *p*_adj_ = .009)
*T. aestivum*	n.s.	Positive relation with mean annual temperature ($\chi_{1}^{2}=28.77$ , *p*_adj_ < .001)	Unimodal relation with soil trophic potential (prefers higher) ($\chi_{1}^{2}=10.34$, *p*_adj_ = .023)

*p*_adj_: *p* values adjusted for multiple comparison by likelihood ratio test. n.s., not significant.

The most significant effect among those of all the environmental predictors was found for soil pH. All the *Tuber* OTUs in this study with the exception of the *T. aestivum* OTU showed significant response to soil pH (Table 4). The *T. aestivum* OTU, in contrast, showed a unimodal response to the soil trophic potential, tending to prefer higher values (Table 4).

**Figure 4:**
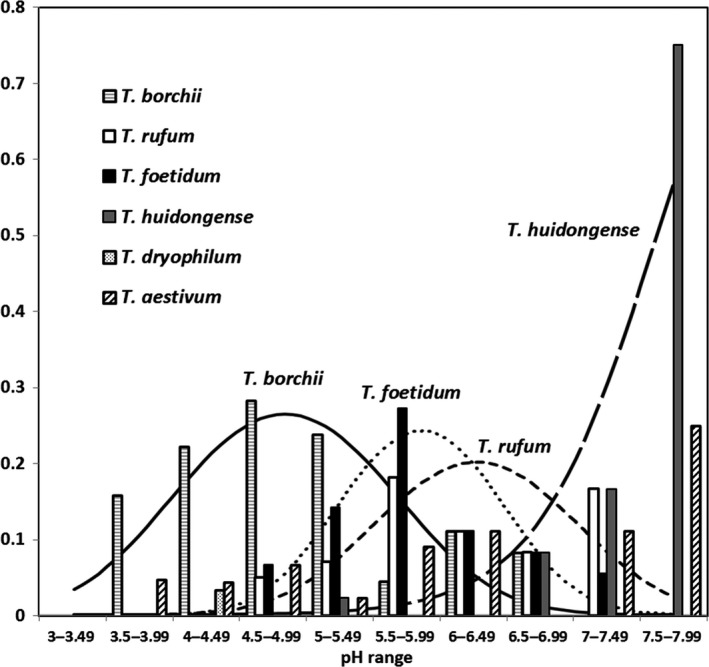
Generalized linear model biplot showing distribution of *Tuber* species along the soil pH gradient, overlaid by normalized counts of positively detected samples for each *Tuber* species in the different soil pH categories. Distributions predicted by the model are shown for *T. borchii* (solid line), *T. foetidum* (dotted line), *T. rufum* (short‐segment broken line), and *T. huidongense* (long‐segment broken line)

Significant effects detected using generalized linear models match the patterns suggested in the CCA biplot (Figure 3), namely a preference for high pH among *T. huidongense*,* T. rufum*, and *T. foetidum* OTUs; a preference for low pH in the case of *T. borchii* OTU (Figure 4); and a preference for higher mean annual temperature of the *T. aestivum* OTU. The effect of *Tilia* spp. host species occurrence was not confirmed when modeling the host species effects on individual OTUs. *Tuber borchii, T. rufum,* and *T. foetidum* OTUs showed unimodal relationships to pH; *T. huidongens*e OTU has a linear and monotonic relationship to soil pH; and *T. aestivum* showed no significant response to soil pH (Figure 4, Table 5).

**Table 5:** Generalized linear model analysis of distribution along soil pH gradient of operational taxonomic units (OTUs) representing different *Tuber* species. Successfuly determined optimum pH and corresponding 95 % confidence intervals are given in bold

	OTU				
	*T. borchii*	*T. rufum*	*T. foetidum*	*T. huidongense*	*T. aestivum*
Model selection (*p* values)					
Model with pH	.0229	.0002	.0052	<.0001	.1736
Model with 2nd‐order polynomials of pH	.0005	.0029	<.0001	n.s.	n.s.
Fitted model summary					
Response type	Quadratic	Quadratic	Quadratic	Linear	–
Explained variation (%)	5.8	19.4	20.8	40.6	–
*F*	8.9	11.8	15.0	30.4	–
*p*	.0002	<.0001	<.0001	<.0001	–
**Optimum pH**	**4.76**	**6.36**	**5.90**	–	–
**95% Confidence interval**	**4.25‐5.18**	**6.00–7.89**	**5.63–6.37**	–	–
Tolerance	0.81	0.76	0.61	–	–

Fitting the generalized linear model with the soil pH predictor expressed as a second‐order polynomial with the logit link function, it was possible to determine the explained variation for all major OTUs (i.e., those detected more than three times), except that *T. aestivum* showed no significant relationship to soil pH (Table 5). *Tuber borchii's* pH optimum was the lowest among the three *Tuber* OTUs with unimodal response. The 95% confidence interval of pH optimum for *T. borchii* OTU did not overlap with the intervals of the other two OTUs (*T. foetidum* and *T. rufum*). In contrast, the confidence intervals of the pH optima for the *T. rufum* and *T. foetidum* OTUs showed a large overlap, indicating that these two species prefer soils with similar pH. Inasmuch as the *T. huidongense* OTU did not exhibit a unimodal relationship with soil pH, it was not possible to identify its pH optimum and associated confidence intervals. It was obvious, however, that the optimum was probably much higher than that for the three OTUs already cited above and showing unimodal response to soil pH (Figure 4).

## 4 Discussion

### 4.1 Incidence of *Tuber* spp. among the sampling sites

Nearly 41% of our samples scored positively for *Tuber* spp. when using genus‐specific PCR primers targeting the β‐tubulin gene. Although our sampling strategy might have caused the abundance of the *Tuber* spp. at the landscape level to be overestimated, this number is nevertheless unexpectedly high and indicates that this group of fungi occurs relatively frequently in central European woodlands. Although not directly comparable (percentage of field sites vs. percentage of root samples), the high abundance of *Tuber* spp. recorded in this study is somewhat in disagreement with the results of Bonito, Brenneman, and Vilgalys (2011), who reported frequency of *Tuber* spp. OTUs in ectomycorrhizae of *Carya ilinoinensis* to be ca 10%–15%. Parádi and Baar (2006) reported the genus *Tuber* as being dominant among 12 “types” of ectomycorrhizal fungi associated with flooded willow in the Netherlands, the percentage of *Tuber* ectomycorrhizae among all mycorrhizal root tips being 29%–50%. At only up to 9%, however, the percentage of ectomycorrhizae among all the root tips was relatively low in that particular study.

Compared to the high incidence of various *Tuber* spp., the incidence of *T. aestivum* was much lower among our sampling sites (5.6%), with most of the positive detections aggregated in the warmest parts of the sampled region. This indicates the particular environmental constraints of this species.

### 4.2 OTU delineation and comparison with earlier ascocarp records from the region

In total, this study detected eight different *Tuber* OTUs in Czech soils by specific PCR. Two of these (*T. indicum and T. oligospermum*) were each found only in a single sample (Table 1). This points to a higher α‐diversity at landscape level than that reported by Bonito Brenneman, et al. (2011), who found only four *Tuber* OTUs at five hardwood sites (albeit with a smaller geographical spread than in our sampling design), but it agrees quite well with previous records from the Czech Republic that are based on ascocarp collections (eight species at country level).

The OTU most frequently detected in our soils was that corresponding to *T. borchii*, constituting 52% of all legible DNA sequences (Table 1). This OTU is well supported by phylogenetic analysis and shows relatively high similarity with the best GenBank hits. Such a high incidence of this OTU is interesting, because it has traditionally been regarded as a rare species, even though it had indeed been described previously from the Czech Republic (Klika, 1926; Vacek, 1948; Valda, 2009). As reported previously, the rarity of previous detection may be due to its inconspicuousness or absence of fructification (Bonito, Brenneman, et al., 2011; Parádi & Baar, 2006). In general, this species is reported as being widely distributed throughout Europe (Riousset, Chevalier, & Bardet, 2001) and that is consistent also with our data.

*Tuber foetidum* was the OTU second most frequently detected in our study. It had previously been recorded only once in the Czech Republic (personal herbarium of S. Valda, Kokořínsko Landscape Protected Area, Mělník, Czech Republic). Although generally *T. foetidum* is considered to be a very rare species (Riousset et al., 2001), our data suggest that it is not particularly rare (at least not as soil mycelium), but probably it is neglected because of its rare fructification.

*Tuber rufum* and *T. huidongense* OTUs are poorly separated on the basis of the β‐tubulin gene sequence. This may be due to high genetic variability of *T. rufum* (Iotti et al., 2007), which is reflected also in the very high standard deviation of the sequence similarities in relation to GenBank best hits (Table 1). At the same time, *T. huidongense* is phylogenetically very close to *T. rufum* (Bonito et al., 2010), that, too, may contribute to the fuzzy separation between the two OTUs in our phylogenetic analysis. *Tuber rufum* was previously collected in the Czech Republic (Vacek, 1947a,b, 1948, 1948, 1950; Valda, 2009), and its relatively high incidence in our soils is thus not particularly surprising. *Tuber huidongense* has not heretofore been reported from the Czech Republic, so our molecular detection is the first record of this species from the region. It is an economically important species that is marketed in large quantities in southwestern China (Wan et al., 2016). The corresponding OTU detected in our analyses has relatively low sequence similarities with the best GenBank hits, reaching just 96%. This possibly indicates a genotype of the species indigenous to Europe or its close relative rather than the Asian genotypes of *T. huidongense*.

Low similarity with the best GenBank hit was noted also for the *T. indicum* OTU, possibly for the same reasons as in the case of the *T. huidongense* OTU stated above. *Tuber indicum* (hitherto unreported from the Czech Republic) is an unwanted, introduced competitor species that may constitute a serious threat to European trufficulture (mainly focused on *Tuber melanosporum* production) posing severe economic and ecological consequences (Bonito, Trappe, et al., 2011). Because of its relatively low similarity to reference sequence GU979181, our record may well represent a heretofore undescribed (or not yet sequenced) indigenous fungus relative of *T. indicum* and not the aggressive invader itself.

The *T. oligospermum* OTU was detected only once in our study, although this species has already been described from the Czech Republic (Valda, 2009). The *T. dryophilum* OTU is newly detected in the Czech Republic, but this species has already been recorded, albeit infrequently, in other European countries (Riousset et al., 2001).

Surprisingly, *Tuber excavatum*, commonly recorded in the field, including in the Czech Republic, and usually accompanying *T. aestivum* (Klika, 1927; Vacek, 1948; Riousset et al., 2001; personal observations of M. Gryndler) were not detected in our molecular survey even though the primers tubtubf and elytubr have efficiently amplified this species previously (Gryndler, Soukupová et al., 2013). Therefore, we establish that this species is comparatively rare relative to the other *Tuber* spp. detectable by our molecular screening.

Still other truffles known on the basis of ascocarp records to inhabit the territory of the Czech Republic were not detected in our molecular survey, including *T. fulgens* (Vacek, 1950; Valda, 2009), *T. mesentericum*,* Tuber maculatum*,* T. regianum* (Valda, 2009), *Tuber nitidum* (Klika, 1926; Vacek, 1950), *Tuber scruposum* (Vacek, 1948), and *T. rapaeodorum* (Vacek, 1947b, 1948, 1950). In total, including the recently reported OTUs, 16 taxa belonging to the *Tuber* genus have now been recorded from the Czech Republic.

### 4.3 Effects of environmental conditions

One of the most striking and novel observations of our survey was ecological niche separation of the different *Tuber* species along the soil pH gradient. The association of the *T. huidongense* OTU with high soil pH is particularly interesting, but no comparable literature data on pH preferences of *T. huidongense* are currently available. The preference observed in our study of *T. borchii* for moderately acidic soils is in agreement with its previously reported tolerance of soils with pH values down to 5.5 (Zambonelli, Iotti, Giomaro, Hall, & Stocchi, 2002). This tolerance to moderately acidic soils may explain why this species is so common in the Czech Republic and in Europe, because large areas in the region have previously been acidified by human activities (Verheyen, Bossuyt, Hermy, & Tack, 1999).

In addition to soils, climatic factors are also presumed significantly to influence the distribution of *Tuber* spp. Detailed information on optimal temperature and precipitation values for *T. aestivum* has been summarized by Stobbe et al. (2013). According to the cited work, the ecological optimum of the species is at mean annual temperature of ca +9°C, mean winter temperature of ca +1°C, and annual precipitation of ca 755 mm. Mean annual and winter temperatures across the sampled sites are 1–2°C lower than those described optima (see Table S1 for details), explaining why *T. aestivum* was significantly associated with warmer sites within our sampling site selection. Perceptible association of *T. aestivum* with soils showing higher soil trophic potential accords with previous results reported by Gryndler, Soukupová et al. (2013). They had observed the same when the properties of the soil colonized by *T. aestivum* were compared with adjacent soil lacking this fungus.

Significant correlation of host tree identity with the incidence of certain *Tuber* OTUs is interesting. This indicates a perceptible preference of *Tuber* spp. for specific hosts under natural conditions, although this preference is generally considered to be rather weak (Gryndler, 2016). Weakness of the host–fungus correlation is also the most likely reason for the inconsistency of results obtained by both CCA and the generalized linear models. In general, CCA explained only about 6.3% of the data set variability, thereby indicating that further (unrecorded) factors play important roles in truffle ecology.

Although significant, the amount of variation in the data set explained by all the tested predictors was generally low. This indicates that factors other than those examined in this study are probably important and should be considered in future studies. For example, the presence of other ectomycorrhizal fungi as competitors (Zambonelli, Iotti, Rossi, & Hall, 2000), history of the land use (including application of fungicides), and visitation of localities by potential vectors of truffle spores (wild boars) should be considered (Piattoni, Ori, Amicucci, Salerni, & Zambonelli, 2016).

### 4.4 Methodological considerations

Only two false‐positive detections were encountered when using the tubtubf and elytubr primers developed by Zampieri et al. (2009). This points to the excellent robustness of those primers in processing environmental samples. The authors of the primers had checked this previously using a number of negative controls (including closely related *Terfezia* sp. and *Choiromyces* sp.) and always with negative results. With respect to false‐positive detection, the primers tubtubf and elytubr were more reliable in detecting the truffles in the field root samples compared to the primers designed by Bertini et al. (1999), who had not performed extensive testing for robustness against false positives and only verified their PCR products by sequencing. This was the reason why we preferred the primers by Zampieri et al. (2009) over those proposed by Bertini et al. (1999). The only false positives we encountered using the primers tubtubf and elytubr were detections of *Helvella ephippium,* a member of a fungal genus which is close to the genus *Tuber* (a member of the sister family Helvellaceae in the order Pezizales) and may thus share a similar β‐tubulin gene sequence with *Tuber* spp. These results indicate that closely related fungi could still be co‐amplified and thus the sequencing of positive amplicons is considered inevitable.

A problem of the primers specific to *Tuber* spp. published by both Zampieri et al. (2009) and Bertini et al. (1999) is that they do not detect all the *Tuber* spp. with the same efficiency. Primers tubtubf and elytubr produced relatively faint amplification signals for *T. indicum, T. macrosporum, T. brumale, T. oregonense, T. gibbosum* and, unfortunately, also *T. aestivum* (Zampieri et al., 2009; Figure 1). In our hands, however, detection of the latter species failed completely (unpublished results, see also Table 2 for the summary). As we were particularly interested in detecting *T. aestivum*, we had to use species‐specific primers for this particular species instead of relying on the genuswide primers. In spite of extensive testing of such specific primer set (Gryndler et al., 2011), we nevertheless recorded two cases of false‐positive detection: *Trechispora invisitata* and *Sphaerosporella* sp. Whereas *Sphaerosporella* (Pezizales, Pyronemataceae) is relatively close to *Tuber* spp., being a member of the same order, *Trechispora* is a phylogenetically distant genus belonging to Basidiomycota (Trechisporales, Hydnodontaceae). The length of PCR product similar to *T. invisitata* was ca 600 bp (data not shown), whereas the length of positive amplicon from *Tuber aestivum* is very close to 500 bp. This false‐positive signal could thus be clearly distinguished already by agarose electrophoresis. This was not the case for the *Sphaerosporella* sp.; however, because it yielded an amplification product with length equal to 550. Also in this case, therefore, a need to sequence the PCR product seems inevitable if one is to sort out false positives from the data set.

Furthermore, the primers used for specific detection of *T. aestivum* produced a high proportion of low‐quality (possibly mixed) amplicons (55%). The reason for this is not at all clear. It cannot be explained by high variability of the ITS region sequence, which is very homogeneous across the various specimens belonging to this species (Gryndler et al., 2011; supplementary materials). Inasmuch as this primer pair also amplifies ITS of *Tuber mesentericum*, however, co‐occurrence of the two *Tuber* species may actually render the amplicon illegible by direct Sanger sequencing. This can be resolved either by restriction analysis of the amplicon (as in our case) or by massively parallel sequencing of the amplicons that would actually sort out much of the uncertainty associated with both the genus‐ and species‐specific primers.

Therefore, we confirmed that the primers developed by Zampieri et al. (2009) specifically to amplify DNA of *Tuber* spp. offer a very robust and particularly useful approach to detecting true truffles in the environment, even though some limitations must be taken into account. First, we confirm the findings of Zampieri et al. (2009) that the primers are not equally sensitive for all the *Tuber* spp. This was the case for *T. aestivum*. In our work, this species had to be detected using a separate primer pair. Second, the specificity of the genus‐specific primers used is not absolute, and the positive amplification signal must always be verified by sequencing the PCR products to exclude false‐positive results. It must be stated here, however, that the specificity of the genus‐specific primers is very high. These produced just two false positives among 322 samples.

## 5 Conclusions

Our results show first and foremost a relatively high incidence of truffles (*Tuber* spp.) within the central European landscape, with *T. borchii* being the species most frequently recorded in the Czech Republic (Table 1). Having said that, we need to re‐emphasize that we a priori preselected the environmental conditions of the sampling sites. That means the sampling design may have somewhat confounded the general message by overestimating the incidence of truffles in the studied region. Our results also clearly demonstrate ecological niche separation of the different *Tuber* species, with *T. borchii* preferring more acidic soils than *T. foetidum*,* T. rufum*, and *T. huidongense* (Figure 4), and the incidence of *T. aestivum* being restricted to the warmest parts of the studied region. Surprisingly, as compared to soil and climatic determinants, there was comparably little evidence for strict host specificity among the different *Tuber* species.

Molecular tools used in this work successfully detected eight OTUs corresponding to true truffles (genus *Tuber*). This number includes two OTUs (*T. indicum* and *T. huidongense*) which have not yet been reported from the Czech Republic. Six species detected by the PCR assays described here had been recorded previously as ascocarps, and another eight truffle species are known from the Czech Republic only from ascocarp records. Thus, we demonstrate a significant overlap of the molecular profiling with the previously recorded list of true truffles while adding two more species not yet reported from this geographical region and demonstrating the usefulness of molecular screening as an unbiased and widely adoptable tool for studying the ecology of hypogeous and edible ectomycorrhizal fungi such as truffles.

*Tuber aestivum* and *T. borchii* are the two most economically important truffle species detected in the Czech Republic. Whereas *T. aestivum* is a species traditionally much appreciated for its culinary value in many European countries, perhaps with the Czech Republic as an exception due to legal restrictions on collecting and marketing this species (Streiblová et al., 2010), *T. borchii* has gained increased attention only in the last two decades. In comparison with the highly prized *Tuber magnatum* and *T. melanosporum*,* T. borchii* has obviously a much wider ecological niche, has low host specificity, and is highly competitive with other mycorrhizal fungi. Thus, *T. borchii* is potentially easier to cultivate (Zambonelli et al., 2002). In addition, our study confirms the tolerance of *T. borchii* for moderately acidic soils, and this may contribute to explaining its widespread occurrence in the central European landscape, affected as it is by human activities (fertilization, cropping, more recently also acid rains) over many centuries.

Moreover, climate changes presently occurring in central Europe may favor *T. borchii* if cold and humid autumns will became typical for this region in place of the previous freezing and dry autumn weather (Salerni, Perini, & Gardin, 2014). This makes *T. borchii* a promising candidate for future trufficulture in the region, offering the use of native germplasm and thus avoiding introgression of alien species and/or genotypes to the region.

## Conflict of Interest

None declared.

## Data Accessibility

All the DNA sequences have been submitted to GenBank, and their accession numbers are listed in Table 1. All raw data (soil properties and climatic parameters, excluding the precise GPS coordinates of the individual sampling sites) are made available as electronic supplement (Table S2) to this paper.

## Author Contributions

MG devised the study. VŠ and KN collected the samples and conducted the chemical analyses and PCR. HH processed the DNA sequencing. MG, PŠ, and JJ analyzed the data and wrote the manuscript.
