# Supplementary material for: Truffle biogeography—A case study revealing ecological niche separation of different Tuber species
Source: Ecol Evol. 2017 May 7;7(12):4275–88. doi: 10.1002/ece3.3017 (PMC5478065; doi:10.1002/ece3.3017)
Supplement: Supplementary file 1 [file ECE3-7-4275-s001.docx]

**Supplementary Table S1 Gryndler et al 2017**

Descriptive statistics and value ranges of climatic parameters and soil properties among the sampled sites.

|  | Mean annual temperature (°C) | Mean winter temperature (°C) | Annual precipitation (mm) | Precipitation balance (mm) | Calcium (mg/kg) | Conductivity (µS/cm) | pH | Trophic potential (A655) |
| --- | --- | --- | --- | --- | --- | --- | --- | --- |
| mean | 7.6 | –0.6 | 797 | –21 | 54.0 | 197 | 4.82 | 2.06 |
| minimum | 5.5 | –2.5 | 600 | –250 | 0.2 | 40 | 3.28 | 0.49 |
| maximum | 9.5 | 0 | 1000 | 250 | 1091 | 970 | 7.61 | 8.02 |
| skewness | –0.1137 | –0.7690 | –0.4269 | –0.2200 | 5.4844 | 2.5248 | 1.1487 | 1.4464 |
| mode | 7.5 | –0.5 | 900 | 0 | 45.4 | 150 | 3.81 | 2.37 |

**Supplementary methods Gryndler et al 2017**

Estimation of soil trophic potential

Soil trophic potential was estimated using filtered and autoclaved aqueous soil extracts (1:2, w:v) in the algae growth test. The culture of the green alga *Chlorella kessleri* (strain LARG/1) was grown for 4 days at 25°C under daylight illumination in 200 ml sterile liquid medium containing (per litre of deionized water): NaNO_3_ – 467 mg, Ca(NO_3_)_2_ . 4H_2_O – 59 mg, K_2_HPO_4_ – 31 mg, MgSO_4_ .7H_2_O – 25 mg, Na_2_CO_3_ – 21 mg, FeNaEDTA – 6.7 mg, H_3_BO_3_ – 3.1 mg, MnSO_4_ . 5H_2_O – 2.4 mg, Na_2_WO_4_ . 2H_2_O – 3.5 µg, (NH_4_)_6_Mo_7_O_24_ . 4H_2_O – 8.8 µg, KBr – 12 µg, KJ – 8.3 µg, ZnSO_4_ . 7H_2_O – 28.7 µg, CdCl_2_ . 2.5H_2_O – 11.5 µg, CoCl_2_ . 6 H_2_O – 11.9 µg, CuSO_4_ . 5H_2_O – 12.5 µg, NiCl_2_ . 6H_2_O – 12 µg, K_2_Cr_2_O_7_ – 2 µg, NaVO_3_ – 1.2 µg, Al_2_(SO_4_)_3_ – 17 µg.

The culture was then centrifuged for 5 min at 180 × g, aseptically re-suspended in 200 ml sterile deionized water, and subjected to 2 days of mineral starvation under daylight illumination at 25°C. After the starvation period, the culture was washed once with sterile deionized water and diluted to reach the absorbance at 655 nm equal to 0.180 (corresponding to the concentration of 4 × 10^5^ cells per ml). This suspension was used as inoculum in the assay.

Five ml of the soil extract (soil re-suspended in water for 20 min, then centrifuged at 3000 × g for 30 min and filtered through paper filter) was autoclaved at 121°C for 25 min and 100 µl aliquots were distributed to 96-well transparent serological plate with flat bottoms. Four wells were used per one sample of the soil extract (analytical replicates). Further, deionized water (blank) and serial dilutions of the above cultivation medium (dilutions 1:2, 1:9 and 1:29) were used as standards, with six analytical replicates each. Each well was then supplied with 25 µl of algal inoculum, initial absorbance at 655 nm was measured, and samples were incubated for 7 days at 25°C, illumination 3000 lux (cool-white fluorescent tubes), 100% relative humidity and 5% CO_2_ in the atmosphere. Thereafter, the absorbance at 655 nm was measured, initial absorbance values were subtracted, and mean absorbance increments were calculated as a measurement of the trophic potential.
